# Sample Size Requirements for Studies of Treatment Effects on Beta-Cell Function in Newly Diagnosed Type 1 Diabetes

**DOI:** 10.1371/journal.pone.0026471

**Published:** 2011-11-10

**Authors:** John M. Lachin, Paula L. McGee, Carla J. Greenbaum, Jerry Palmer, Mark D. Pescovitz, Peter Gottlieb, Jay Skyler, Type 1 Diabetes Trial Network

**Affiliations:** 1 The Biostatistics Center, The George Washington University, Rockville, Maryland, United States of America; 2 Benaroya Research Institute, Seattle, Washington, United States of America; 3 Department of Medicine, University of Washington, Seattle, Washington, United States of America; 4 Department of Surgery and Microbiology/Immunology, Indiana University Medical Center, Indianapolis, Indiana, United States of America; 5 Barbara Davis Center for Childhood Diabetes, University of Colorado Health Sciences Center, Denver, Colorado, United States of America; 6 Diabetes Research Institute, University of Miami School of Medicine, Miami, Florida, United States of America; Genentech Inc., United States of America

## Abstract

Preservation of 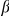-cell function as measured by stimulated C-peptide has recently been accepted as a therapeutic target for subjects with newly diagnosed type 1 diabetes. In recently completed studies conducted by the Type 1 Diabetes Trial Network (TrialNet), repeated 2-hour Mixed Meal Tolerance Tests (MMTT) were obtained for up to 24 months from 156 subjects with up to 3 months duration of type 1 diabetes at the time of study enrollment. These data provide the information needed to more accurately determine the sample size needed for future studies of the effects of new agents on the 2-hour area under the curve (AUC) of the C-peptide values. The natural log(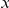), log(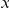
+1) and square-root 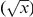 transformations of the AUC were assessed. In general, a transformation of the data is needed to better satisfy the normality assumptions for commonly used statistical tests. Statistical analysis of the raw and transformed data are provided to estimate the mean levels over time and the residual variation in untreated subjects that allow sample size calculations for future studies at either 12 or 24 months of follow-up and among children 8–12 years of age, adolescents (13–17 years) and adults (18+ years). The sample size needed to detect a given relative (percentage) difference with treatment versus control is greater at 24 months than at 12 months of follow-up, and differs among age categories. Owing to greater residual variation among those 13–17 years of age, a larger sample size is required for this age group. Methods are also described for assessment of sample size for mixtures of subjects among the age categories. Statistical expressions are presented for the presentation of analyses of log(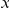
+1) and 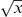 transformed values in terms of the original units of measurement (pmol/ml). Analyses using different transformations are described for the TrialNet study of masked anti-CD20 (rituximab) versus masked placebo. These results provide the information needed to accurately evaluate the sample size for studies of new agents to preserve C-peptide levels in newly diagnosed type 1 diabetes.

## Introduction

Type 1 diabetes results from a T-cell mediated progressive autoimmune destruction of the insulin secreting pancreatic 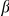-cells [1], and numerous therapeutic targets and agents have been proposed to ameliorate this process [2] based on a growing understanding of the underlying mechanisms. The measurement of C-peptide in response to a stimulus provides a valid and reliable measure of the effects of therapy on residual 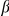-cell function [3], the preferred stimulus being a mixed-meal tolerance test [4], as recognized in the recent FDA guidance on drug development in newly diagnosed type 1 diabetes [5]. Unfortunately, published reports from recently completed trials generally do not present the measures of residual variation and other quantities needed to guide sample size determination for future trials. The best available data [3] were based on a pooling of data from prior published and unpublished studies in subjects with a wide range of diabetes duration, heterogeneous methods of collection and assays, and limited follow-up.

The Type 1 Diabetes Trial Network, established by the National Institute of Diabetes, Digestive and Kidney Diseases, recently conducted two therapeutic trials in recent onset type 1 diabetes. Herein the available data from these studies are used to describe the effects of different transformations on the distributional properties (e.g. normality) of the C-peptide values, and to evaluate the sample size (or power) for a new study.

## Methods

### Subjects

The anti-CD20 study [6] enrolled 87 subjects, 81 meeting the intention-to-treat criteria (52 rituximab, 29 placebo). The results showed that rituximab significantly preserved 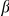-cell function at the primary 12-month outcome visit [6]. The analyses herein employ the 30 placebo treated subjects who completed the 12 month examination, including an additional placebo subject who had been excluded from the intention-to-treat cohort because placebo infusions (double masked) were halted owing to a safety alert.

The MMF/DZB study [7] included 126 subjects randomly assigned to either mycophenolate mofetil alone or in combination with daclizumab, or a control group, who were followed for up to 2 years. Therapy was terminated for futility in the spring of 2008 by the external Data and Safety Monitoring Board after observing virtually no differences in C-peptide levels among the treatment groups. Further, since the two treated groups in the MMF/DZB study [7] were no different from placebo, the data from the 126 MMF/DZB study subjects were pooled with the 30 anti-CD20 placebo control group subjects as the basis for the analyses herein.

### Methods and Procedures

The MMF/DZB and anti-CD20 studies enrolled male and female subjects between ages 8–45 within 100 days of diagnosis of Type 1 diabetes who had at least one islet autoantibody and peak stimulated C-peptide 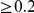 pmol/ml. Stimulated C-peptide values were obtained during a 2 or 4 hour mixed-meal tolerance test (MMTT) [4] conducted at baseline, 3, 6, 12, 18 and 24 months. Only the 2-hour data are employed herein. Over 5 minutes, participants ingested the Boost liquid oral dietary supplement (mixed meal, Nestlé HealthCare Nutrition, Inc.) dosed relative to body weight. Basal (fasting) plasma samples were collected 10 minutes prior to the meal (−10), just prior to the time of ingestion (0), and at 15, 30, 60, 90 and 120 minutes thereafter. C-peptide levels were measured centrally at the 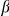-cell function laboratory (Seattle, WA). The primary outcome was the area under the 2-hour curve (AUC) in pmol/ml/120 min computed using the trapezoidal rule. The corresponding “ AUC mean” in pmol/ml is obtained as AUC/120 [3], [4]. Non-measurable timed values were set equal to the lower limit of quantification of the assay before computing the AUC.

### Statistical Considerations

Most C-peptide values will fall between 0 and 1 and the distribution is positively skewed [3]. Thus, scale-contracting transformations were considered. However, the log transformation could introduce negative skewness because 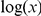 approaches 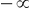 as the value 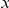 approaches zero. This can be corrected by using 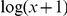 [8]. The square-root transformation compresses the distribution of values 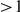 and slightly expands the distribution of values between 0 and 1. Both the MMF/DZB and anti-CD20 studies pre-specified that the primary analyses would employ the 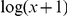 values.

Commonly, the primary analysis compares the mean of the C-peptide values between treatment groups after a period of treatment such as 12 or 24 months. With normally distributed errors, the most powerful test is an Analysis of Covariance (ANCOVA) adjusting for the baseline C-peptide value [9], and other baseline factors such as age and sex as previously recommended [3]. Algebraically, this is equivalent to an analysis using the change from baseline when adjusted for the baseline value [10]. Herein the analyses of the follow-up AUC mean values from the 2 hours of the MMTT are presented using the combined data from the two studies with an adjustment for study (MMF/DZB versus anti-CD20) and treatment group so as to account for any chance differences among studies and groups.

ANCOVA assumes normally distributed residuals with constant variation over the range of C-peptide values (homoscedasticity). The residuals were obtained from the regression of each subject's raw or transformed variables on age, sex, study and treatment group within study. The distribution of the residuals was evaluated using quantile-quantile plots [11]. The Shapiro-Wilks test [12] assessed departures from normality. White's test [13] assessed the assumption of homoscedasticity (constant error variances among subjects).

For each transformation 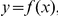 the mean values and confidence limits are presented using the inverse transformation applied to the mean of the transformed values, 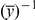 , and the corresponding confidence limits. Thus, for an analysis using 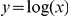, the inverse mean is the geometric mean 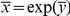. For an analysis using 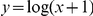, the inverse mean is the geometric-like mean 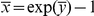. For an analysis using 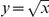, the inverse mean is 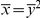.

An analysis using the log-transformed values is readily described in terms of geometric means and a percentage difference between groups. The final Results sub-section on Statistical Computations shows how an analysis using the 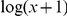 transformed values can also be described as a ratio of geometric means, and an analysis using the 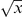 values can be described as a difference in ordinary means, both in units of pmol/ml. That section also derives expressions that can be employed to compute the standard errors and confidence limits for the inverse transformed means following an analysis with either the 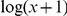 or 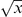 transformations.

### Ethical Statement

The anti-CD20 and/or MMF/DZB study protocols and consent documents were approved by the IRB of Benaroya Research Institute (Seattle), Children's Hospital Los Angeles, Columbia University, The George Washington University, Indiana University, Institut Feur Diabetes (Munich), Joslin Diabetes Center, San Raffaele University (Milan), Stanford University, University of California San Francisco, University of Colorado, University of Florida, University of Miami, University of Minnesota, University of Pittsburgh University of Texas Southwestern Medical School, University of Toronto, and the University of Washington. Each institution participated in one or in both studies that generated the data on which this report is based. All consents were obtained in writing.

## Results

### Distribution Properties

**Figure 1:**
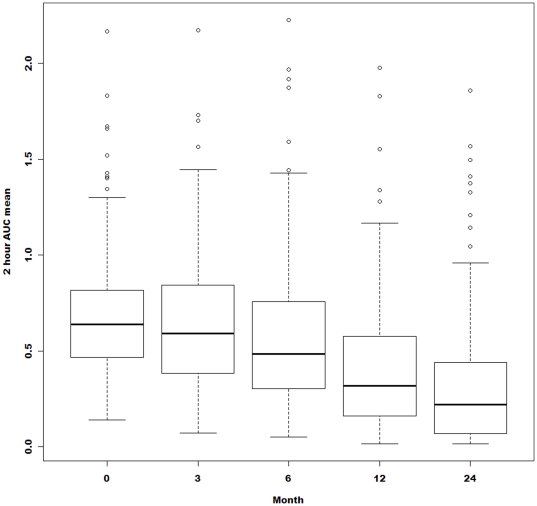
Box plots showing the quartiles, the 5th and 95th percentiles (whiskers) and extreme values to the minimum and maximum (o) of the raw AUC mean C-peptide values for all subjects at baseline and at months 6, 12, 18 and 24.

The characteristics of the subjects in the two studies were comparable owing to the similar eligibility criteria (Table 1). Of the complete cohort, 152 (97%) were evaluated at 12 months and 118 (76%) at 24 months, the latter owing to early termination of the MMF/DZB study. Figure 1 also presents box and whisker plots of the values over time which show that the distributions are strongly positively skewed with an elongated right tail.

**Table 1:** Characteristics of the MMF/DZB study cohort and the Anti-CD20 Control Group.

	MMF/DZB cohort	Anti-CD20 control cohort	Combined Cohort
	N = 126	N = 30	N = 156
Age (years)	18.7 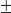 9.6	17.3 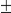 7.8	18.4 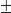 9.3
8–12 years	37 (29%)	8 (27%)	45 (29%)
13–17 years	39 (31%)	13 (43%)	52 (33%)
18 years and older	50 (40%)	9 (30%)	59 (38%)
Sex (% male)	76 (60%)	18 (60%)	94 (60%)
Race (% white)	118 (94%)	29 (97%)	147 (94%)
Ethnicity (% Non-Hispanic)	119 (94%)	27 (90%)	146 (94%)
HbA1c (%)	7.6 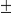 1.3	7.1 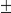 1.3	7.5 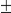 1.3
Total insulin dose/kg	0.37 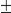 0.21	0.38 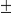 0.22	0.38 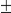 0.20
AUC Mean C-peptide (pmol/ml)	0.70 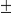 0.33	0.74 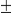 0.37	0.71 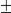 0.34
Time since diagnosis (days)	77 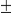 19	83 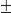 19	78 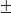 19
Number (%) of positive Autoantibodies*			
0	2 (2%)	1 (3%)	3 (2%)
1	21 (17%)	7 (23%)	28 (18%)
2	43 (34%)	11 (37%)	54 (35%)
3	60 (48%)	11 (37%)	71 (46%)
# Subjects with 2 h MMTT at each visit			
Baseline	126	30	156
Month 12	122	30	152
Month 24	94	24	118

*Among ICA, GAD65, and ICA512.

**Figure 2:**
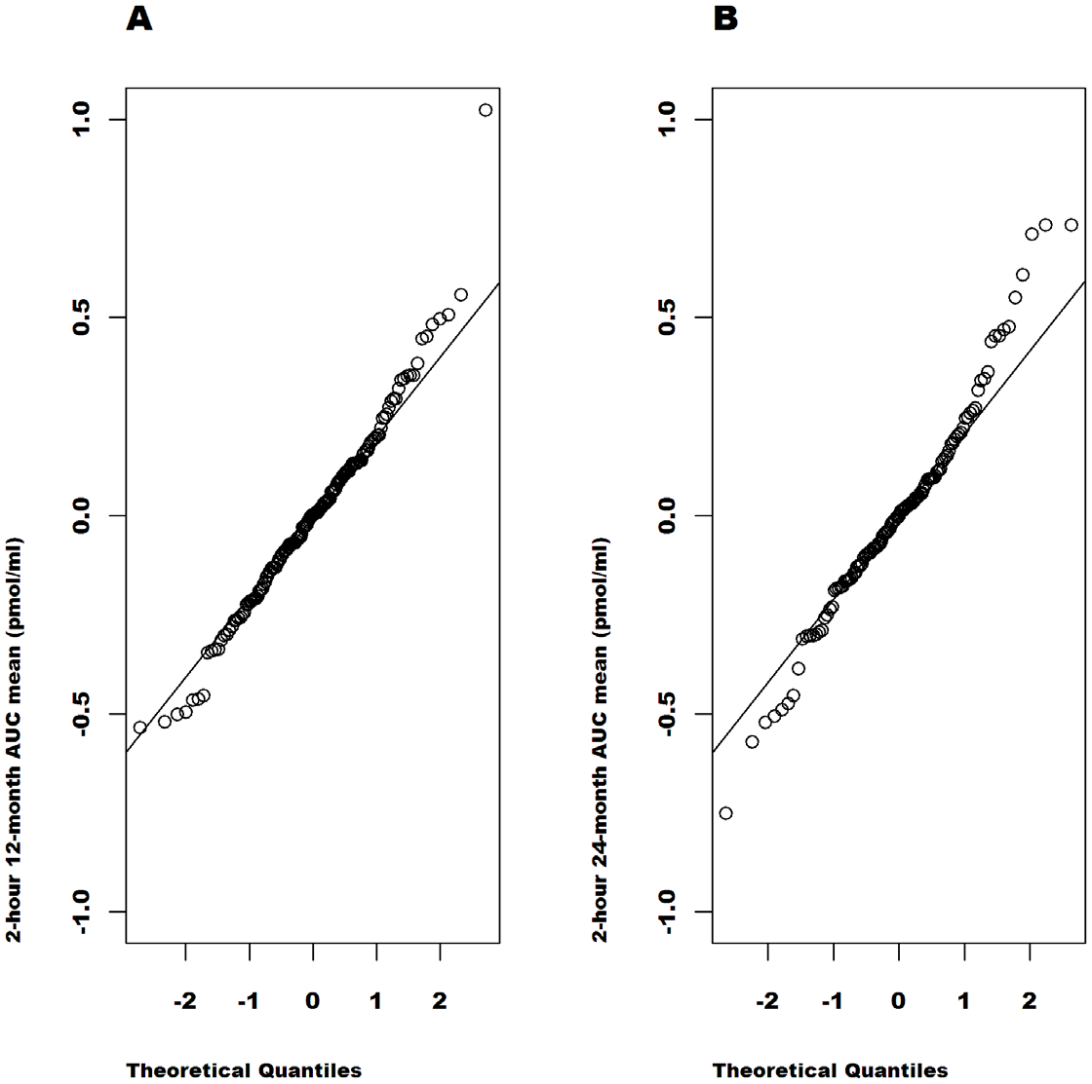
Q-Q plots of the model residuals for untransformed AUC mean values from the combined studies separately at 12 (A) and 24 months (B). Based on the distribution of the residuals in an ANCOVA model adjusted for the baseline untransformed C-peptide value, age, sex, study and treatment group.

**Figure 3:**
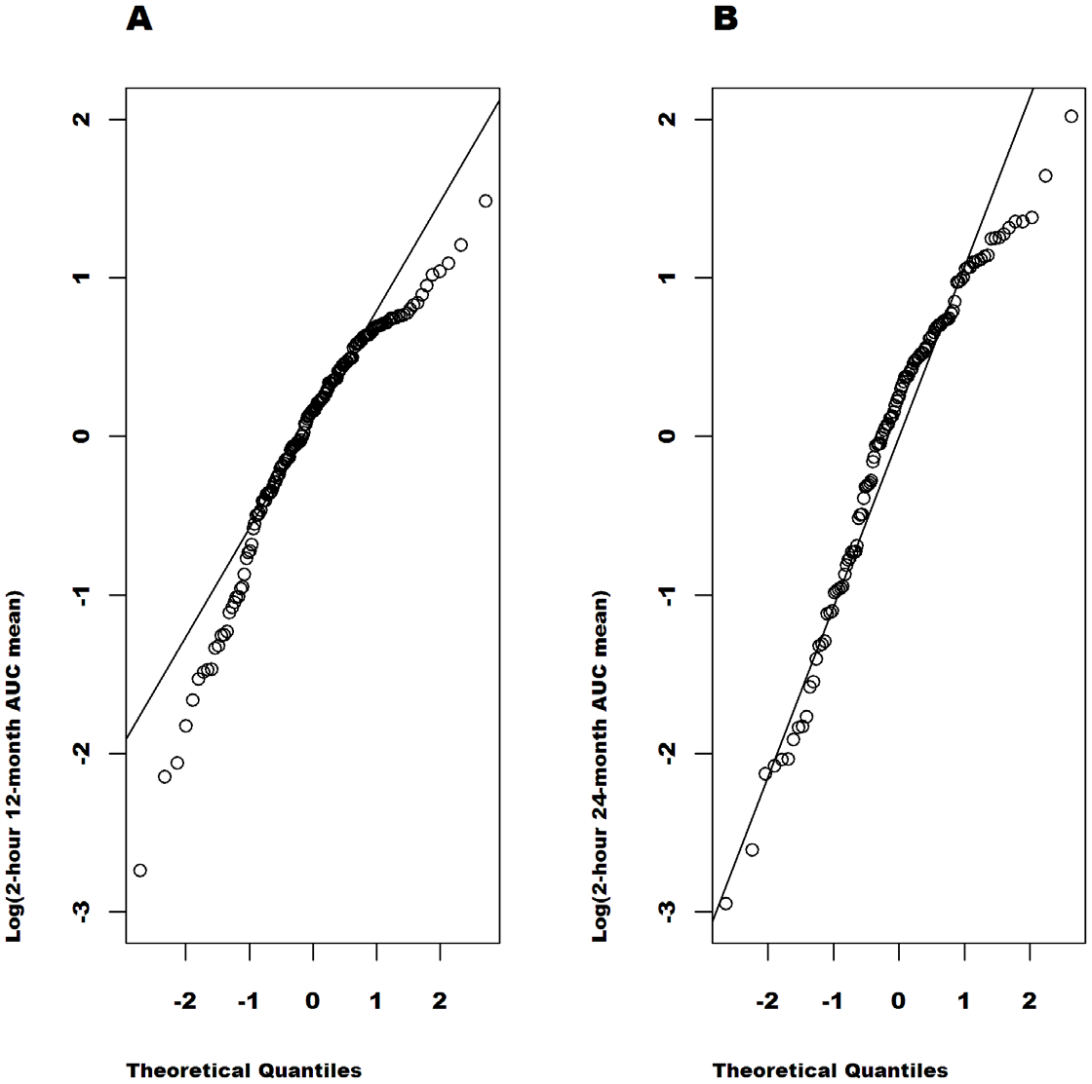
Q-Q plots of the model residuals for natural log-transformed AUC mean values from the combined studies separately at 12 (A) and 24 months (B). Based on the distribution of the residuals in an ANCOVA model adjusted for the log baseline C-peptide value, age, sex, study and treatment group.

**Figure 4:**
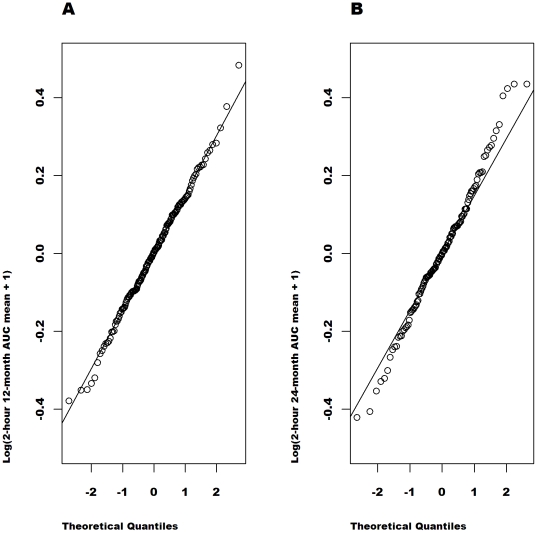
Q-Q plots of the model residuals for 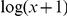 transformed AUC mean values from the combined studies separately at 12 (A) and 24 months (B). Based on the distribution of the residuals in an ANCOVA model adjusted for the baseline log (C-peptide+1) value, age, sex, study and treatment group.

**Figure 5:**
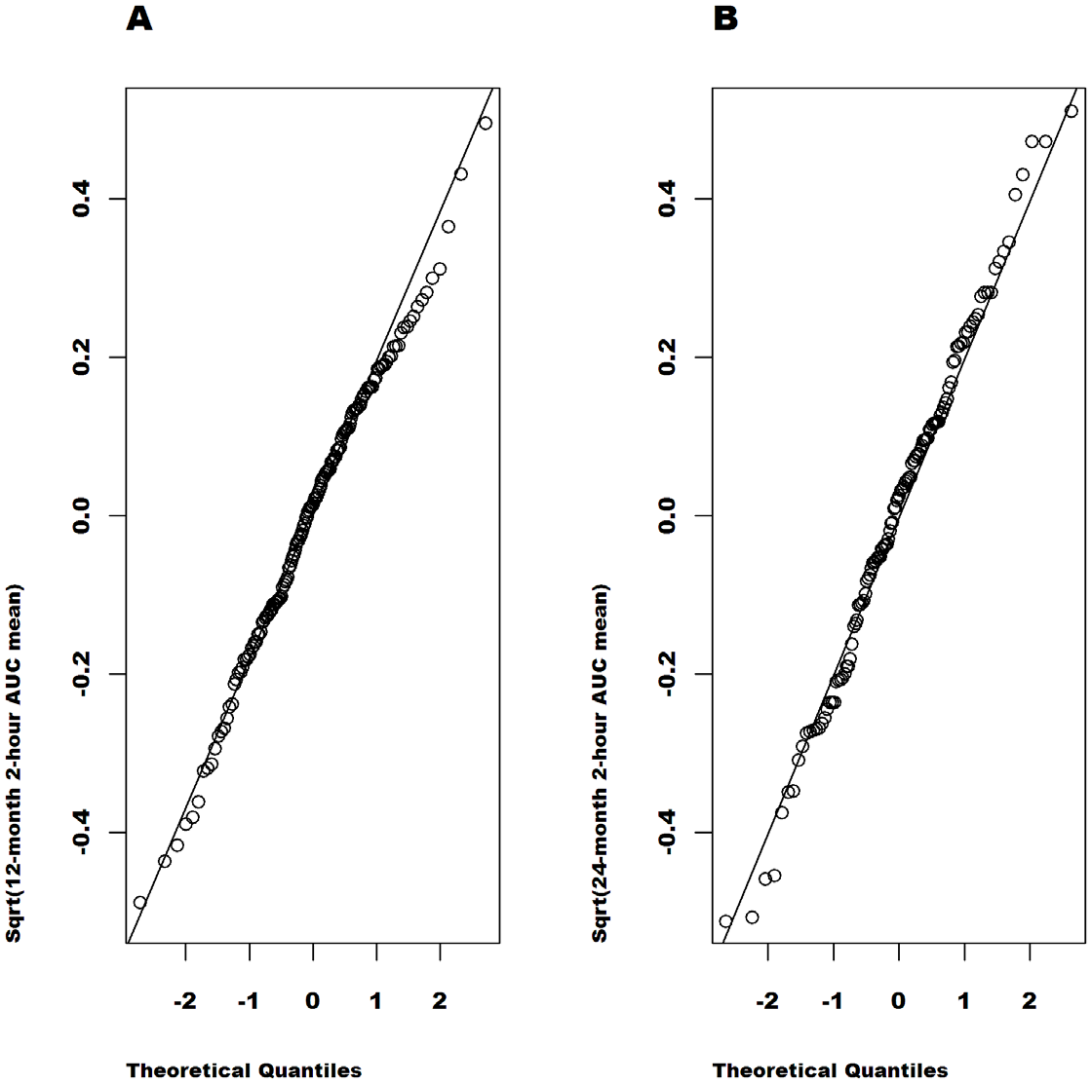
Q-Q plots of the model residuals for square-root transformed AUC mean values from the combined studies separately at 12 (A) and 24 months (B). Based on the distribution of the residuals in an ANCOVA model adjusted for the baseline square-root C-peptide value, age, sex, study and treatment group.

Figures 2, 3, 4, 5 show the quantile-quantile (Q-Q) plots of the residuals from an analysis of the raw and the transformed values at 12 and at 24 months adjusted for the baseline value, age, sex, study (MMF/DZB versus anti-CD20) and treatment group within study. These plots compare the empirical quantiles (the dots) versus those expected from a normal distribution (the diagonal line). The observed data is normally distributed when the observed values fall directly on the line. For both the 12 and 24-month data, the raw AUC mean values show the most severe departures from normality with a distribution that is far too peaked and with right skewness. The ideal normal distribution would in fact have longer symmetric tails that would fall outside of the range of the observed values. The 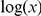 transformation expands the left tail, but too much so, generating left skewness and does not correct for the peakedness. The 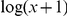 and the 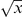 transformation both provide a more symmetric and less peaked distribution relative to the ideal normal.

Table 2 presents the Shapiro-Wilks test and White's test results. The Shapiro-Wilks test of normality showed significant departures from normality for the raw AUC mean values and the 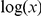 values at both 12 and 24 months, but not the 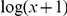 and 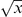 values (results not shown). At 12 months, White's test of homoscedasticity suggested somewhat greater heteroscedasticity (non-constant error variance) for the 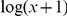 values, comparable to that for the raw values, but each measure had similar results at 24 months.

**Table 2:** 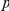
-values for the Shapiro-Wilks test of departures from normality and White's test of homoscedasticity for analyses of the AUC mean C-peptide at 12 and 24 months without or with transformations after adjustment for age and sex at baseline, study and treatment group, and the baseline value on the same scale (i.e. non-transformed or transformed the same as the follow-up values).

	Month 12		Month 24	
	Shapiro-Wilks	White	Shapiro-Wilks	White
*AUC mean (x)*	0.0102	0.0065	0.0413	0.4599
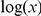	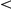 0.0001	0.6646	0.0033	0.2844
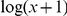	0.9010	0.0193	0.4907	0.2820
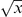	0.4810	0.2122	0.8418	0.1987

These analyses suggest that the distributional assumptions for an ANCOVA test of means at 12 or 24 months are adequately met using either the 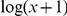 or using the 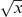 transformation. The 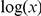 values appear to have substantial left skewness but this is attributable to a small number of values close to zero. In practice, this transformation may also be considered. Thus, in the following we describe the assessment of sample size using all of these approaches.

### Sample Size Computations

The power of the test of means depends on the absolute difference between groups on the chosen scale, the sample size and the residual variation as measured by the standard deviation (SD) or root mean square error (RMSE) 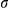. For a given type I error probability 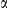, residual RMSE (SD) 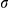, and fraction of subjects assigned to the treated group 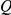, equation (1) of the sub-section on Statistical Computations provides the sample size 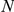 needed to provide desired power 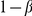 to detect a difference 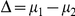 in the mean values on the chosen scale in the treated group and control groups [14]. If no transformation was employed then 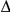 is the difference in the untransformed or raw mean values. If a transformation was employed then 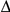 is the difference in the means of the transformed values.

Table 3 presents the quantities needed to compute sample size or power for non-stratified analyses without or with a transformation separately for an analysis at 12 months and at 24 months. The table also presents the relevant quantities within three age strata shown in Table 1 since the mean C-peptide values and standard deviation vary according to age, as described below. The baseline transformed and inverse mean is also presented for reference. While the trial properties could be described in terms of the change from baseline, as described in the methods, from [10] in terms of changes from baseline would have the exact same power as an analysis of the follow-up visit values when both analyses are also adjusted for baseline. Thus, for simplicity, computations are described in terms of the month 12 or 24 values, not the changes from baseline.

**Table 3:** Quantities required to assess sample size or power using an analysis of the untransformed or transformed AUC (pmol/ml) values at 12 or 24 months, along with the baseline mean values (transformed and inverse).

A. 12 months, combined cohort control group								
	Baseline Mean		Transformed		Inverse		Residual SD	
	Transf.	Inverse	Mean	90% limit	mean	90% limit	RMSE	90% limit
*AUC mean (x)*								
Overall	0.71	0.71	0.42	0.40	0.42	0.40	0.239	0.259
Age 8–12 y	0.63	0.63	0.29	0.26	0.29	0.26	0.174	0.206
Age 13–17 y	0.75	0.75	0.35	0.31	0.35	0.31	0.250	0.291
Age 18+ y	0.73	0.73	0.57	0.53	0.57	0.53	0.244	0.288
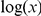								
Overall	−0.46	0.63	−1.24	−1.32	0.29	0.27	0.755	0.818
Age 8–12 y	−0.59	0.56	−1.64	−1.78	0.19	0.17	0.741	0.876
Age 13–17 y	−0.35	0.71	−1.38	−1.55	0.25	0.21	0.927	1.079
Age 18+ y	−0.47	0.63	−0.80	−0.88	0.45	0.41	0.477	0.549
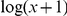								
Overall	0.52	0.68	0.33	0.31	0.39	0.36	0.154	0.167
Age 8–12 y	0.47	0.60	0.24	0.22	0.27	0.25	0.120	0.142
Age 13–17 y	0.55	0.73	0.29	0.26	0.34	0.30	0.175	0.204
Age 18+ y	0.52	0.69	0.42	0.40	0.52	0.49	0.147	0.169
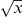								
Overall	0.82	0.67	0.60	0.58	0.36	0.34	0.183	0.198
Age 8–12 y	0.77	0.59	0.50	0.47	0.25	0.22	0.153	0.181
Age 13–17 y	0.85	0.73	0.55	0.51	0.30	0.26	0.215	0.250
Age 18+ y	0.82	0.68	0.71	0.68	0.50	0.46	0.160	0.184

**The one-sided lower 90% confidence limit for the control group mean and the upper 90% limit for the root mean square error (RMSE) are also shown.**

Power depends on the difference between the means on the chosen scale (i.e., without or with transformation), and the smaller this difference the lower the power. Likewise, power depends on the residual RMSE, and the higher the value the lower the power. There is sampling variation in both of these quantities such that the values in a future study could be higher or lower than those observed herein, affecting power. Thus, more conservative estimates of sample size and power are provided using the one-sided lower 90% confidence limit for the mean and the upper 90% limit for the SD. Table 4 then presents sample size computations for an analysis at 12 and 24 months, respectively, using either the non-transformed or transformed data assuming a one-sided test at the 0.05 level, 85% power and a 2∶1 allocation ratio (treated∶control) with no losses to follow-up. These are the design parameters adopted as a template for TrialNet studies; however, additional computations with other design parameters are readily obtained from the equations presented in the sub-section on Statistical Computations. Also, to be conservative, in Table 4 the 90% lower limit for the control group mean and upper limit for the RMSE from Table 3 are employed. For an analysis of the raw AUC mean values, there is no transformation so the difference between groups 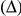 refers to a difference in the original units (pmol/ml). That difference is often specified in terms of a percentage difference, such as 50%. Alternately the difference could be specified in terms of an algebraic difference (subtraction).

**Table 4:** Sample sizes* for two groups with 2∶1 allocation (Q = 2/3) to treatment versus control needed to provide 85% power to detect either a 50% difference, or an absolute difference of 0.2 pmol/ml, using either the raw or transformed data for an unstratified analysis at 12 or at 24 months.

Analysis at 12 months								
	Inverse Mean (pmol/ml)		Transformed Mean					
	Control	Treated	Control	Treated	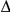	RMSE 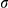	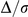	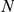
50% difference								
AUC mean 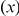	0.4	0.6	–	–	0.20	0.259	0.77	53.0 (54)
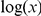	0.27	0.41	−1.32	−.90	0.42	0.818	0.51	131.0 (132)
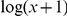	0.36	0.54	0.31	0.43	0.12	0.167	0.72	57.7 (60)
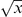	0.34	0.51	0.58	0.71	0.13	0.198	0.66	73.1 (75)
0.2 pmol/ml Difference								
AUC mean 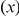	0.4	0.6	–	–	0.20	0.259	0.77	53.0 (54)
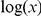	0.27	0.47	−1.32	−0.76	0.56	0.818	0.68	70.1 (72)
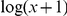	0.36	0.56	0.31	0.44	0.13	0.167	0.78	47.4 (48)
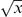	0.34	0.54	0.58	0.73	0.15	0.198	0.76	54.5 (57)

*All computations are for a one-sided test at the 0.05 level with no adjustment for losses to follow-up. In all cases the 90% limits for the control group mean and the SD are used as the parameter values in equation (1). The exact N is provided as well as that rounded up to the nearest integer satisfying the 2∶1 allocation fractions.

For example, in Table 4 the estimated untransformed mean at 12 months in the control group is 0.4 pmol/ml. A 50% increase yields a mean of 0.6 in the treated group with 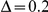 pmol/ml. With RMSE 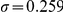, the standardized difference is 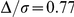 with resulting 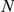 = 53.0, rounded up to 54, the next highest number divisible by 3. Alternately, the difference to be detected could have been specified as an algebraic difference of 0.2 pmol/ml, rather than a 50% increase, yielding the same result in this case. However, in other cases shown, a 50% difference may not be equivalent to a difference of 0.2 pmol/ml.

For sample size calculations where a transformation will be employed, it is important to note that the analysis, and thus the means and 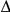 (and the RMSE 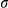), must be specified in terms of the transformed values. However, the meaningful difference to be detected is generally specified in terms of the inverse means in pmol/ml. Consider, for example, detecting a 50% difference using the 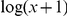 transformation. The control group mean of the 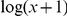 values is 0.31 log(pmol/ml+1). The inverse control group geometric-like mean is 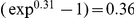 pmol/ml. A 50% difference yields a value of 0.54 pmol/ml in the treated group with a corresponding 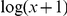 value of 0.43 log(pmol/ml+1). Compared to the mean value of 0.31 log(pmol/ml+1) in the control group, this yields 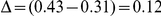. When employed in equation (1) with RMSE 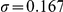, the resulting 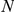 = 57.7 that is rounded to 60.

Alternately, the difference could be specified as an algebraic difference in the inverse mean values, such as a 0.2 pmol/ml difference in the geometric-like means. In this case the treated group inverse mean would be 0.56 pmol/ml and the corresponding 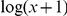 value is 0.44. This yields a 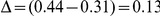 log(pmol/ml+1) and the resulting 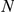 = 47.4 that is rounded to 48. This smaller sample size arises because a 0.2 pmol/ml difference between groups in the inverse means results in a larger 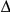 in the transformed values (0.13) than when the effect is specified as a 50% improvement (0.12).

The sample size required to detect a 50% difference is larger using the 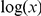 values than the other approaches because its corresponding standardized difference 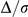 is smaller. The smaller the standardized difference, the larger is the required sample size. Using the 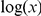 transformation yields 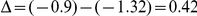 log(pmol/ml) and 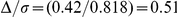, compared to 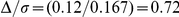 using the 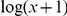.

The sample size required to detect a 50% difference at 24 months is double that needed to detect a 50% difference at 12 months. One reason is that the control group-mean is smaller, due to the progressive loss of C-peptide leading to lower values at 24 than at 12 months, some of which are virtually zero (and still included in the analysis). As a result, a 50% increase in the pmol/ml values results in a slightly smaller difference 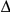 between the transformed means at 24 than at 12 months, except for the 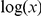 analysis that is unchanged from 12 months. But the main reason is the higher RMSE at 24 months than at 12 months, more so for the 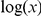, resulting in smaller standard difference values 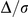 and larger 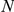 than at 12 months.

However, the sample size needed for a study designed to detect a 0.2 pmol/ml difference between groups at month 24 is about the same as that at month 12, except for the 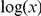. While the RMSE of the 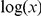 is greater at 24 than 12 months, so also is the mean difference so that the standard difference 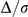 is greater at 24 months, resulting in a smaller sample size requirement at month 24 than at month 12.

The sub-section on Statistical Computations also presents equations for the computation of power for a given 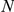 using equation (2) therein, and the computation of the difference 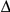 that can be detected with a given level of power with a specific 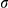 and 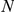.

### Influence of Age

In Table 3, the residual SD values in the 13–17 year age category are substantially higher than those in the other age strata at 12 months but not at 24 months. Thus, a substantially larger sample size would be required for a 12-month study in this age group, or predominantly containing this age group, regardless of whether the treatment effect of interest is stated as a percentage difference or an absolute difference between groups.

Table 3 also shows that the control group mean values at 12 and at 24 months are substantially higher in the 18+ year category than in the other two strata. This indicates that a smaller sample size would be required to detect a given percentage treatment group difference within this age stratum than within other age strata. The table, however, also presents the baseline transformed and inverse means within each age stratum. Compared to the month 12 and 24 values, the baseline values are also higher in the 18+ category than the 8–12 category. This indicates that the C-peptide is declining at a lower rate in the 18+ year category and that a smaller algebraic treatment group difference might therefore be observed, thus requiring a larger sample size.

For illustration, Table 5 presents the sample size calculations for a 12-month study restricted to each of the three age strata using the 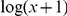 values. To detect a 50% difference, a study in adults would require a much smaller sample size, but to detect a 0.2 pmol/ml difference, a study in children 8–12 y would require a smaller sample size. In both cases, a study restricted to adolescents 13–17 y of age would require the largest sample size.

**Table 5:** Sample sizes* for two groups with 2∶1 allocation (Q = 2/3) to treatment versus control needed to provide 85% power to detect either a 50% difference, or an absolute difference of 0.2 pmol/ml, using an age-stratified analysis of log(x+1) values at 12 months.

Analysis at 12 months using the 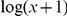 values with three age strata								
	Inverse Mean (pmol/ml)		Transformed Mean					
	Control	Treated	Control	Treated	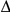	RMSE 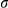	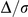	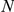
50% difference								
Age 8–12 y	0.25	0.375	0.22	0.32	0.10	0.142	0.70	87.5 (90)
Age 13–17 y	0.30	0.45	0.26	0.37	0.11	0.204	0.54	139.6 (141)
Age 18+ y	0.49	0.735	0.40	0.55	0.15	0.169	0.89	51.8 (54)
0.2 pmol/ml Difference								
Age 8–12 y	0.25	0.45	0.22	0.37	0.15	0.142	1.06	28.3 (30)
Age 13–17 y	0.30	0.50	0.26	0.41	0.15	0.204	0.74	62.0 (63)
Age 18+ y	0.49	0.69	0.40	0.52	0.12	0.169	0.71	51.8 (54)

*All computations are for a one-sided test at the 0.05 level with no adjustment for losses to follow-up. In all cases the 90% limits for the control group mean and the SD are used as the parameter values in equation (1). The exact N is provided as well as that rounded up to the nearest integer satisfying the 2∶1 allocation fractions.

### Age Mixtures

The TrialNet data herein consists of a mixture of subjects within the three age categories as shown in Table 1 that applies to the overall estimates presented in Table 3. However, the mixture of the age groups within a study may differ from that herein, such as for a study that is restricted to adults alone initially, followed by enrollment of adolescents and adults. In this case, a sample size computation could be based on a weighted average of the age specific quantities, with weights equal to the proportion of subjects expected to fall within each age stratum. Details are presented in the sub-section on Statistical Computations.

### The Anti-CD20 Study Results

The anti-CD20 study published results demonstrated significant differences between the rituximab and control group subjects in the primary intent-to-treat analysis of the 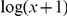 values at one year of follow-up [6]. That single analysis was pre-specified in the study protocol because any post-hoc selected analysis could substantially inflate the type I error probability and lead to biased results. Nevertheless, it is instructive to examine what the study results would have been had a different approach to the analysis been chosen.

Table 6 presents the ANCOVA model adjusted treatment group effect using the untransformed and transformed 12-month C-peptide values. As expected from above, the analysis of untransformed values failed to reach statistical significance, whereas those of the transformed values were each statistically significant. While the 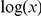 analysis produced the smallest p-value, its F-value was not substantially different from that of the other analyses.

**Table 6:** Analysis of covariance (ANCOVA)* of the difference between the rituximab versus control groups in the distributions of AUC mean values at 12 months of follow-up without and with transformations adjusted for age and sex and the baseline value.

	Rituximab	Control					
	Transformed	Transformed			Standardized	F-	
	mean (95% CI)	mean (95% CI)	Difference	RMSE	Difference	value	p-value
	N = 49	N = 29†					
AUC mean 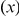	0.604	0.531	0.073	0.248	0.294	1.53	0.11
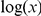	−0.736	−1.096	−0.360	0.613	0.587	6.12	0.008
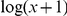	0.445	0.383	0.063	0.141	0.447	3.48	0.03
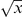	0.737	0.657	0.079	0.161	0.491	4.32	0.021

*In each analysis, the like-transformed (or untransformed) baseline AUC mean value was employed as an adjusting covariate, e.g. 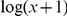 of the baseline value in the analysis of the 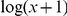 12 month values. The means of the transformed (or untransformed) values within groups, and the difference, are presented along with the root mean square error (RMSE) of those values, the standardized difference, the F-value and one-sided p-value for the effect of treatment.

† These analyses are based on the intention-to-treat cohort that includes 29 placebo-treated subjects who met the defined criteria. The results using the 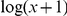 values are identical to those that appeared in the primary study manuscript [6].

The distribution of the raw AUC mean residuals in this study was not as distorted that in the combined cohort data. The distributions of the transformed values were similar to those in the combined cohorts. The Shapiro-Wilks test was again significant for the raw and 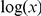 values, but White's test values were similar in the four analyses.

The 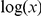 or other transformation may produce different variances between groups, thus violating one of the assumptions of the ANCOVA test [15]. The F-test of equality of the variances of the covariate-adjusted values was highly significant for the 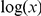 transformed values (p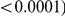 and marginally for the 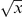 values (p = 0.046), but not the raw values or 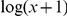 values. In this case, Satterthwaite's test, that allows for unequal variances, yields p = 0.017 for the difference between groups in the 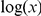 values, and p = 0.019 for the 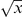 values.

Two additional distribution-free tests of the difference in baseline-adjusted values also provided significant results. A Wilcoxon test (also called a rank transformation analysis [16]) provided one-sided p-values ranging from 0.023 to 0.029; and a test using White's robust information-sandwich estimate of the variance [13] yielded p-values almost identical to those in Table 6 (0.061, 0.008, 0.018 and 0.012, respectively).

Thus, even though the 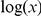 distribution departs from normality, it nevertheless provides a significant difference between groups as did the other transformations. Were the 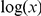 analysis pre-specified as the primary analysis, the result would still be valid, though the test would have less power than one using a more appropriate transformation. Table 7 then shows the inverse means and the relative and absolute differences between groups on each scale. Among the different analyses, the percentage difference in the inverse means was greatest for the 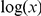 values even though the control group mean was lower. The algebraic differences were similar among the analyses. Table 6 shows the transformed means, the RMSE and the standardized difference for each analysis. The standardized difference that determines power is slightly greater for the 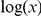 values.

**Table 7:** For each ANCOVA in Table 6 *, the inverse-transformed means (pmol/ml) are presented along with 95% confidence limits, the algebraic difference and the percentage difference.

	Rituximab	Control	Difference	
	Inverse mean	Inverse mean		
	(95% CI)	(95% CI)	Algebraic	%
AUC mean 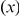	0.60	0.53	0.07	13%
	(0.53, 0.68)	(0.44, 0.62)		
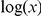	0.48	0.33	0.15	45%
	(0.40, 0.57)	(0.27, 0.42)		
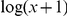	0.56	0.47	0.09	20%
	(0.50, 0.63)	(0.39, 0.55)		
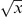	0.54	0.43	0.11	26%
	(0.47, 0.61)	(0.36, 0.51)		

*These analyses are based on the intention-to-treat cohort that includes 29 placebo-treated subjects who met the defined criteria. The results using the 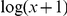 values are identical to those that appeared in the primary study manuscript [6].

### Statistical Computations

This sub-section presents the statistical equations used in the computations presented in the main paper, with additional examples. This includes methods for the calculation of sample size and power; the assessments based on different mixtures of subjects in the three age strata; and the computation of a ratio or difference in the mean levels using either the log(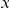
+1) or square root transformations. Throughout, the C-peptide value 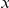 refers to the AUC mean value in pmol/ml.

#### Computation of Sample Size and Power

The equations used to compute sample size and power are widely available, as in [14]. Let 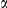 denote the type 1 (false positive) error probability and 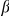 the type II (false negative) error probability. Then 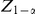 is the critical value for the test statistic at level 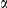, one or two-sided as pre-specified; e.g. 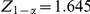 for a one-sided 0.05 level test, 1.96 for a two-sided test. 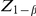 is the quantile corresponding to the desired level of power 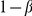, e.g. 1.04 for 85% power. To allow for an unequal allocation, let 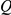 designate the fraction of subjects assigned to the treated group, 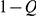 that to control, e.g. 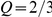 for a 2∶1 randomization to treatment and control. Then let 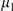 denote the mean of the transformed (or untransformed) values in the treated group and 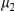 that in the control group, with difference 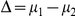. Denote the root mean square error (RMSE) of the transformed values as 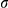. Then the sample size needed to detect the difference 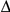 in the transformed values is provided by the equation
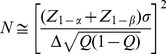
(1)where 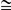 designates approximate equality.

To allow for a fraction (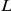) losses-to-follow-up or missing outcome data, then the sample size would inflated by 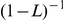. For example, if 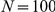 with complete follow-up, then to adjust for 20% losses the sample size would be inflated to yield 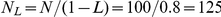.

In some cases, the sample size may be specified from other considerations and it may then be desired to evaluate the power of the study to detect a given difference. In this case, for a given 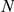 the power is computed from
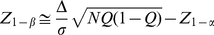
(2)where power = 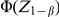 is the cumulative normal fraction at the value 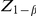, e.g. power = 0.85 for 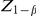 = 1.04. Alternately, the study properties may be specified in terms of a difference 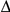 that can be detected with a given level of power with a specific 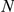
and 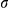, as provided by
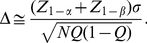
(3)The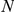 employed in equations (2) and (3) should be the number of evaluable subjects. For example, if the planned 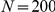, to allow for 10% losses, then the equation should employ 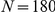.

The above simple equations are approximations to the precise computations using the non-central student's t-distribution for which iterative computations are required. See [14], among many. For 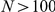 the precise sample size is less than 2% greater than that provided by the above equation (1), and less than 1% for 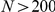.

For example, Table 4 shows that a sample size of 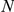 = 60 provides 85% power to detect a 50% difference using the 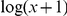 transformed values at 12 months, whereas a sample size of 117 would be required at 24 months. If the study is conducted using N = 60, the power to detect a 50% difference at 24 months could be computed using (2). From Table 4, a 50% difference in a 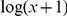 analysis at 24 months would yield a value 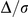 = 0.52. Substituting this quantity along with 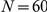 yields 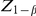 = 0.254 that corresponds to power of 60%.

Alternately, the difference that can be detected with 85% power could be computed from (3) upon substituting 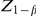 = 1.04 (for 85% power), 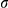 = 0.192 (the RMSE from Table 4) and 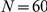 to show that this sample size would provide 85% power to detect a difference 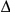 = 0.14 in the 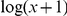 values at 24 months. Adding this amount to the control transformed mean in Table 4 (0.23) yields a treated transformed mean of 0.24+0.14 = 0.38. Taking the inverse transformation of each yields means of 0.27 pmol/ml for the control group and 0.46 pmol/ml for the treated group, or a 70% difference.

#### Age-Averaged Estimation

As described in the text, other studies may comprise a mixture of age categories that differs from that in the TrialNet studies. Denote the fraction of subjects within the three age categories as 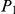 for age 8–12, 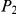 for 13–17, and 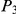 for 18 and older. For a specified difference between groups, that could vary among the age strata, let 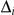 denote the difference to be detected within the 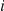
th age stratum, and 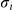 the residual standard deviation within each stratum (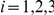). Then, the average expected difference between groups is
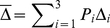
(4)and the average residual variance would be 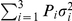. Then sample size would be computed using
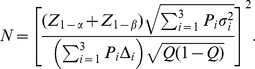
(5)Likewise, the power of the study for a given sample size would be obtained from
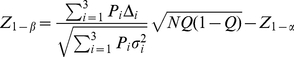
(6)and the average difference that can be detected as
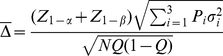
(7)


For example, consider a study designed to detect a 50% difference using the 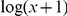 transformed values in an analysis at 12 months that is projected to enroll fractions 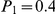, 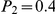, and 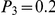. From the quantities specified in Table 3, a 50% increase in the 90% limit of the inverse control group means, the resulting transformed means, and 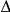 are 
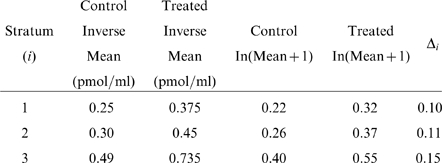



Then the terms needed for the sample size calculation are
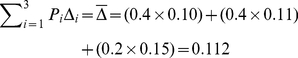
(8)

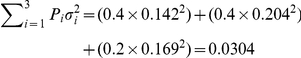


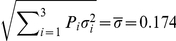



#### Interpretation of log(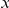) and log(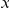
+1) Analyses

For an analysis of the raw values, standard programs will compute the baseline adjusted mean values and their differences. For an analysis on the log scale, taking the exponential function of the baseline adjusted means provides estimates of the geometric means. Programs also provide an estimate of the difference between the means of either the raw values or the log values. In the latter case, taking the exponential function of the difference provides an estimate of the ratio of the geometric means.

However, for an analysis using the log(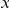
+1) transformation or the square root transformation, programs do not directly provide estimates of the ratio or difference of the corresponding C-peptide mean values on the pmol/ml scale. Herein we show how these estimates can be obtained from other computer program computations.

If the log(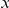
+1) transformation is used as the basis for the analysis of the levels of C-peptide it is useful to summarize the results using the ratio of the geometric means, say 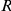, with 95% confidence limits on 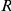. Since 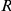 is a ratio with a value of 1 under the null hypothesis, asymmetric confidence limits computed using the log of 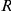 will provide more accurate coverage probability than symmetric limits based on the simple estimated standard error of 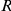 itself. The necessary quantities can be obtained from an analysis of the log(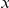
+1) values using a program such as a SAS PROC GLM or PROC MIXED to compute the adjusted means (called LSmeans) and their standard errors.

Let 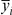 refer to the LSmean of the log(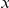
+1) values in the 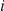
th group 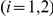. Then 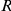 is expressed as
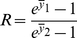
(9)and the log geometric mean ratio 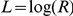 as
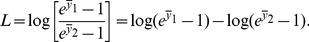
(10)The variances of each mean, say 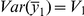 and 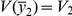, are provided by squaring the standard errors provided by the LSMEANS computation. An additional computation is needed to obtain the covariance of the two.

The LSMEANS output also includes a computation of the difference, i.e. 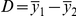, but not the SE of the difference. Thus, an estimate statement is used to obtain the variance (or SE) of the difference between the group LSmeans. Since
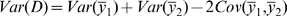
(11)then the covariance, say 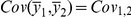, is obtained by subtraction as
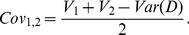
(12)Using the delta method it is then shown that the variance of the log ratio is
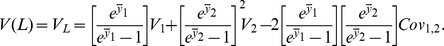
The 95% asymmetric confidence limits on 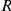 are then obtained as
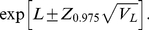
(13)


#### Interpretation of 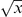 Analyses

If the analysis uses the 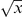 transformation it would be desirable to summarize the results using the difference of the inverse means, say 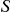, with 95% confidence limits on 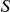, both computed in pmol/ml units. As above, let 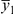 refer to the LSmean of 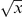 in one group and 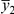 that in the other group, with respective variances 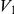 and 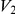 obtained as the square of the standard errors. Then the difference 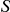 in pmol/ml units is expressed as
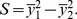
(14)Again using the delta method, the variance of 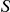 is obtained as
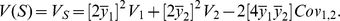
The 95% symmetric confidence limits on 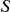 are then obtained as
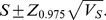
(15)


## Discussion

Analyses of two recently completed TrialNet studies in newly diagnosed type 1 diabetes assess the properties of the stimulated C-peptide levels of a 2-hour Mixed Meal Tolerance Test used to measure 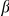-cell function. In general, a transformation is needed to improve the normality of the distribution of values. Among those considered herein, the 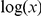 over-corrects, replacing right skewness with left skewness, whereas the 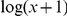 and 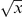 values are more nearly symmetrically distributed. The resulting sample size estimates for an analysis using the 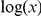 values are greater than using either the raw or 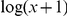 and 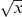 values. The sample sizes using the 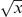 values were slightly greater than those using the 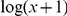 values.

TrialNet pre-specified that the 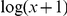 transformation would be used so as to improve the distribution because the majority of the AUC mean values are less than 1 [3]. Another approach to deal with this might be to simply multiply the AUC mean values 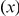 by a constant 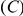, such as multiplying by 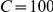 to yield values 100*AUC mean in pmol/(ml/100). Taking the log transformation yields 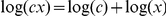. In this case the shape of the distribution of 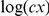 is the same as that of 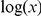 and the properties of the analysis of the 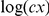 values is the same as that of an analysis of the 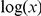 values.

While TrialNet had initially selected the 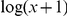 transformation based on its preliminary data, it is possible that preliminary studies of a compound might suggest that a different transformation best captures the effect of treatment on the distribution of values. For example, if a preliminary study suggests that an analysis of the raw values appears to best reflect the treatment group difference, then a sample size calculation using the raw values might be preferred even though, based on the computations herein, a smaller sample size might be computed using a transformation. Likewise, preliminary data from other studies might suggest that a different transformation, like 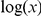, might be preferred, in which case we hope that the data presented herein could be useful for planning future studies.

Sample size computations are shown for an analysis at 12 and 24 months using either a relative (50%) increase or a fixed (0.2 pmol/ml) difference between groups. The 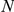 required to detect a fixed difference is principally a function of the residual variation (RMSE) that tends to be greater at 24 than at 12 months, resulting in a larger 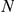 at 24 months. The 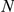 required to detect a relative increase is also a function of the control group mean because a percentage increase from a larger control mean value equates to a larger absolute difference. For example, in Table 4, a 50% increase in the 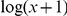 values at 12 months corresponds to a difference of 0.12 pmol/ml versus a difference of 0.10 pmol/ml at 24 months in Table 4, again resulting in a larger 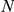 at 24 months.

In practice it might be more appropriate to consider a larger difference between groups at 24 than at 12 months. For example, if an effective treatment actually stabilized the level of C-peptide over 2 years, then owing to the progressive decline in the control group, there should be a larger difference between groups at 24 than at 12 months that would lead to the requirement for a smaller sample size.

The results also depended on age, stratified herein as 8–12 years, 13–17 and 18 and older at diagnosis. The residual variation among those 13–17 years was substantially higher than that in the other age categories, perhaps because they are peripubertal. Thus, methods are described to compute sample size for a study with specific planned fractions of subjects in these age categories.

It may also be prudent to consider different effect sizes within the age strata. Comparing the inverse mean values within the age strata at 12 months versus 24 months (Table 3), the rate of decline in those 18 and above is less than that in the other categories. Thus, a treatment that stabilizes the level of C-peptide over 2 years would have a smaller treatment effect among those 18 and above because the control group would be falling at a lower rate. This could readily be addressed by using a smaller difference in this age category when conducting an age specific computation as shown in the sub-section on Statistical Computations.

The TrialNet anti-CD20 study showed a statistically significant beneficial effect (p = 0.02) of rituximab versus placebo on the pre-specified 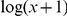 C-peptide at 12 months [6]. Additional analyese presented herein show that the differences in the 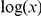 and 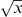, but not the raw, values were also statistically significant, more so for the 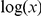. While the 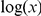 violated the common variance assumption based on White's test being significant (Table 2), other non-parametric or robust tests not requiring that assumption were also significant. Such a test might be preferred if it is decided to use the 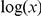 values in the analysis of a study.

The optimal transformation may also differ for other methods of analysis. For example, a secondary analysis of the anti-CD20 study assessed the difference between groups in the average rate of decline (or slope) in the C-peptide values over time [6]. Biologically, a constant percentage decline per year in C-peptide would be expected [3], corresponding to the rate of decline in 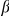-cell mass. This constant percentage decline implies that the slope of the 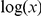 values is constant over time, or that the 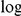 C-peptide is a linear function of time with coefficient 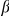, and the percentage change in C-peptide per year is estimated as 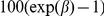. Neither an analysis of the 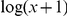 nor 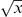 values would have this interpretation. On this basis, the 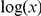 values were employed in the slope analysis presented in the published report [6]. This analysis used a random coefficient model [17] allowing a unique rate of change in 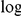 C-peptide over time for each subject with an estimate of the mean slope within each treatment group. The mean percentage decline in the rituximab group was significantly less than that with placebo (38 versus 56% per year, p = 0.027). However, had the analysis been done using the raw, 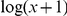 or 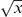 values, none would have approached significance (p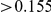 for all).

It should also be noted that there are many other possible transformations that might be employed. Among the most general is the family of Box-Cox power transformations [18], [19] that can often transform a set of quantitative values to a near normal distribution. Such transformations are often used to promote a strongly linear association among variables on the transformed scales. Rarely, however, are such transformations used for inferences about the underlying mean values, as is the focus herein.

Clearly, the results herein largely apply to a population of subjects recruited in North America. Whether they apply to studies conducted in other populations is unknown. However, the distributions of C-peptide values obtained from a cross sectional study of the properties of a mixed meal versus glucagon stimulation test conducted in North America were similar to those of an identical study conducted in Europe [4], despite the fact that different central laboratories were employed in each study. Further, it is remarkable that consistent patterns of change in C-peptide over time have been observed in the control groups of studies conducted different populations [6], [7], [20]–[23].

In conclusion, these TrialNet studies support the need to employ a transformation in the analysis of C-peptide values over time in therapeutic studies of new onset type 1 diabetes. The patterns of variation differ after 12 months and 24 months, and among age categories. However, it is possible to fine-tune the design of a study in a manner that allows for these factors.

## Supporting Information

Supplement S1 Members of the Type 1 Diabetes Trial Network.
(DOCX)

## References

[1] Atkinson MA, Maclaren NK (1994). The pathenogenesis of insulin-dependant diabetes mellitus.. N Engl J Med.

[2] Ludvigsson J. The role of immunomodulation therapy in autoimmune diabetes.. J Diabetes Sci Technol.

[3] Palmer JP, Fleming GA, Greenbaum CJ, Herold KC, Jansa LD (2004). C-peptide is the appropriate outcome measure for type 1 diabetes clinical trials to preserve beta-cell function: report of an ADA workshop, 21–22 October 2001.. Diabetes.

[4] Greenbaum CJ, Mandrup-Poulsen T, McGee PF, Battelino T, Haastert B (2008). Mixed-meal tolerance test versus glucagon stimulation test for the assessment of beta-cell function in therapeutic trials in type 1 diabetes.. Diabetes Care.

[5] Food and Drug Administration (2008). Guidance for Industry Diabetes Mellitus: Developing Drugs and Therapeutic Biologics for Treatment and Prevention.. http://www.fda.gov/downloads/Drugs/GuidanceComplianceRegulatoryInformation/Guidances/ucm071624.pdf.

[6] Pescovitz M, Greenbaum C, Krause-Steinrauf H, Becker D, Gitelman S, Goland R, Gottlieb P, Marks J, McGee P, Moran A, Raskin P, Rodriguez H, Schatz D, Wherrett D, Wilson D, Lachin J, Skyler JS (2009). Preservation of Beta-Cell Function by B-Lymphocyte Depletion with Rituximab in Patients with New Onset Autoimmune Diabetes.. N Engl J Med.

[7] Gottlieb PA, Quinlan S, Krause-Steinrauf H, Greenbaum C, Wilson D, Rodriguez H, Schatz D, Moran A, Lachin JM, Skyler JS (2010). Failure to Preserve beta-cell function with Mycophenolate Mofetil and Daclizumab Combined Therapy in patients with new onset Type 1 Diabetes.. Diabetes Care.

[8] Bartlett, MS (1947). The Use of Transformations.. Biometrics.

[9] Neter J, Kutner MH, Nachtsheim CJ, Wasserman W (1996). Applied linear statistical methods. 4th ed.

[10] Laird N (1983). Further comparative analyses of pretest-posttest research designs.. The American Statistician.

[11] Thode HC (2002). Testing for normality.

[12] Shapiro SS, Wilk MB (1965). An Analysis of Variance Test for Normality (complete samples).. Biometrika.

[13] White H (1980). A heteroskedasticity-consistent covariance matrix estimator and a direct test for heteroskedasticity.. Econometrica,.

[14] Lachin JM (1981). Introduction to sample size determination and power analysis for clinical trials. Control. Clin.. Trials.

[15] Wolfe R, Carlin JB (1999). Sample-size calculation for a log-transformed outcome measure. Control. Clin.. Trials.

[16] Conover WJ, Iman RL (1981). Rank Transformations as a Bridge Between Parametric and Nonparametric Statistics.. The American Statistician.

[17] Demidenko E (2004). Mixed Models: Theory and Applications.

[18] Box GEP, Cox DR (1964). An analysis of transformations.. J Roy Statist Soc Ser B.

[19] Sakia RM (1992). The Box-Cox transformation technique: a review.. The Statistician.

[20] Herold KC, Hagopian W, Auger JA, Poumian-Ruiz E, Taylor L (2002). Anti-CD3 monoclonal antibody in new-onset type 1 diabetes mellitus.. N Engl J Med.

[21] Keymeulen B, Vandemeulebroucke E, Ziegler AG, Mathieu C, Kaufman L (2005). Insulin needs after CD3-antibody therapy in new-onset type 1 diabetes.. N Engl J Med.

[22] Raz I, Elias D, Avron A, Tamir M, Metzger M (2001). *β*-cell function in new-onset type 1 diabetes and immunomodulation with a heat-shock protein peptide (DiaPep277): a randomized, double-blind, phase II trial.. Lancet.

[23] Ludvigsson J, Faresjö M, Hjorth M, Axelsson S, Chéramy M (2008). GAD Treatment and Insulin Secretion in Recent-Onset Type 1 Diabetes.. N Engl J Med.

